# Supplementary material for: Field-tunable toroidal moment in a chiral-lattice magnet
Source: Nat Commun. 2021 Sep 9;12:5339. doi: 10.1038/s41467-021-25657-6 (PMC8429646; doi:10.1038/s41467-021-25657-6)
Supplement: Supplementary file 1 — Supplementary Information [file 41467_2021_25657_MOESM1_ESM.pdf]

Field-tunable toroidal moment in a chiral-lattice magnet

Supplementary Information

Lei Ding[1,*], Xianghan Xu[2,*], Harald O. Jeschke[3], Xiaojian Bai[1], Erxi Feng[1], Admasu Solomon Alemayehu[2], Jaewook Kim[2], Feiting Huang[2], Qiang Zhang[1], Xiaxin Ding[4], Neil Harrison[4], Vivien Zapf[4], Daniel Khomskii[5], Igor I. Mazin[6,†], Sang-Wook Cheong[2,†], Huibo Cao[1,†]

[1]*Neutron Scattering Division, Oak Ridge National Laboratory, Oak Ridge, TN 37831, USA*
[2]*Rutgers Center for Emergent Materials and Department of Physics and Astronomy, Piscataway, NJ 08854, USA*
[3]*Research Institute for Interdisciplinary Science, Okayama University, Okayama 700-8530, Japan*
[4]*Los Alamos National Laboratory, Los Alamos, New Mexico 87545, USA*
[5]*II. Physikalisches Institut, Universität zu Köln, Zülpicher Straße 77, D-50937 Köln, Germany*
[6]*Department of Physics and Astronomy, George Mason University, Fairfax, VA 22030, USA*

Contents

S.1 Crystal structure and magnetic structure determination of $BaCoSiO_4$ **2**

S.2 Magnetization characterization **7**

S.3 Density functional theory calculations **8**

S.4 Theoretical modeling **11**

*These authors contributed equally to this work.
†Email: imazin2@gmu.edu, sangc@physics.rutgers.edu, caoh@ornl.gov

S.1 Crystal structure and magnetic structure determination of $BaCoSiO_4$

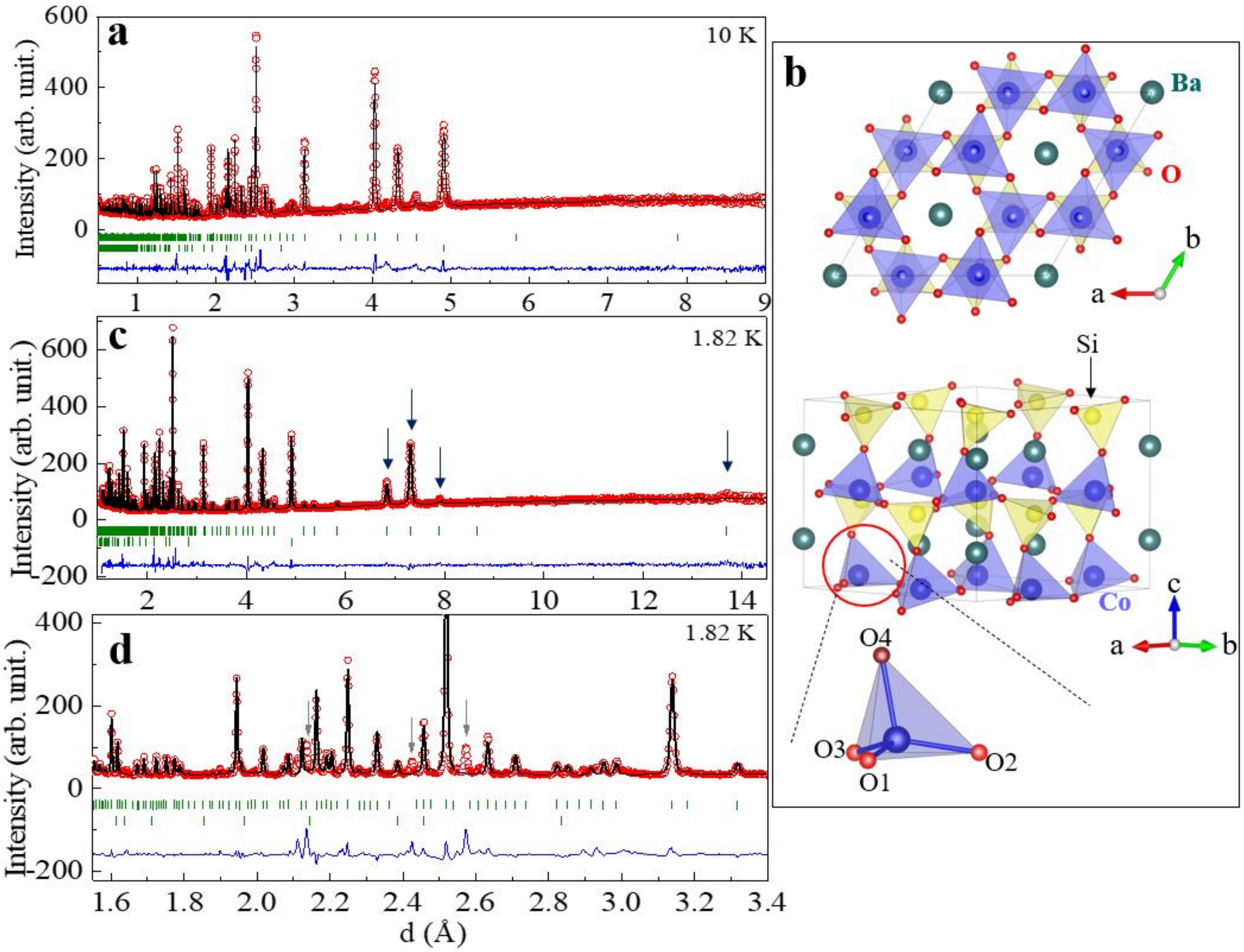

Figure S1: **a**, Rietveld refinement results of neutron diffraction data collected at 10 K. Open red circles and solid black line represent experimental and calculated intensities, respectively. Solid blue line at the bottom of the panel shows the difference between them. The upper green tick marks stand for the positions of the Bragg reflections while the lower marks denote the impurity phase Ba1.1CoO_3 (weight fraction: $8.59(5)\%$). **b**, Schematic representation of the crystal structure. The distorted CoO_4 tetrahedron is emphasized by demonstrating the considerable off-center of the Co atoms. **c**, Rietveld refinement of neutron diffraction data collected at 1.8 K using the magnetic space group $P6_3$. The black arrows mark the magnetic reflections. **d** Enlarged figure **c** to present the refinement at low d-space. The gray arrows show the unindexed contribution from impurity phases.

The structure of $BaCoSiO_4$ measured by neutron powder diffraction at 10 K above T_N has the space group $P6_3$, consistent with the reported one from the single crystal X-ray diffraction at room temperature [1]. The Rietveld refinement here adopts the reported crystal structure as a starting structural model [1]. Background was described by linear interpolation of selected points in the pattern. The profile function of the powder time-of-flight neutron diffraction data was described by a convolution of a pseudo-Voigt function with a pair of back-to-back exponential, implemented by the FULLPROF SUITE program. The Rietveld refinement of the neutron diffraction pattern is shown in Fig. S1a and the corresponding structural parameters are tabulated in Table S1. As shown in Fig. S1b, Co^{2+} ions are tetrahedrally coordinated by oxygen forming spin trimers in the ab plane, which are bridged by the adjacent SiO_4 tetrahedra. The spin trimer layer is stacked alternately with the SiO_4 tetrahedra layers along the c axis. The CoO_4 tetrahedron is considerably distorted with a large off-center of Co atoms and one relatively distant (Co-O4) bond ($d_{Co\text{-}O4}$ = 1.994 Å) and three smaller bond lengths ($d_{Co\text{-}O1}$ = 1.946 Å, $d_{Co\text{-}O2}$ = 1.944 Å, $d_{Co\text{-}O3}$=1.938 Å).

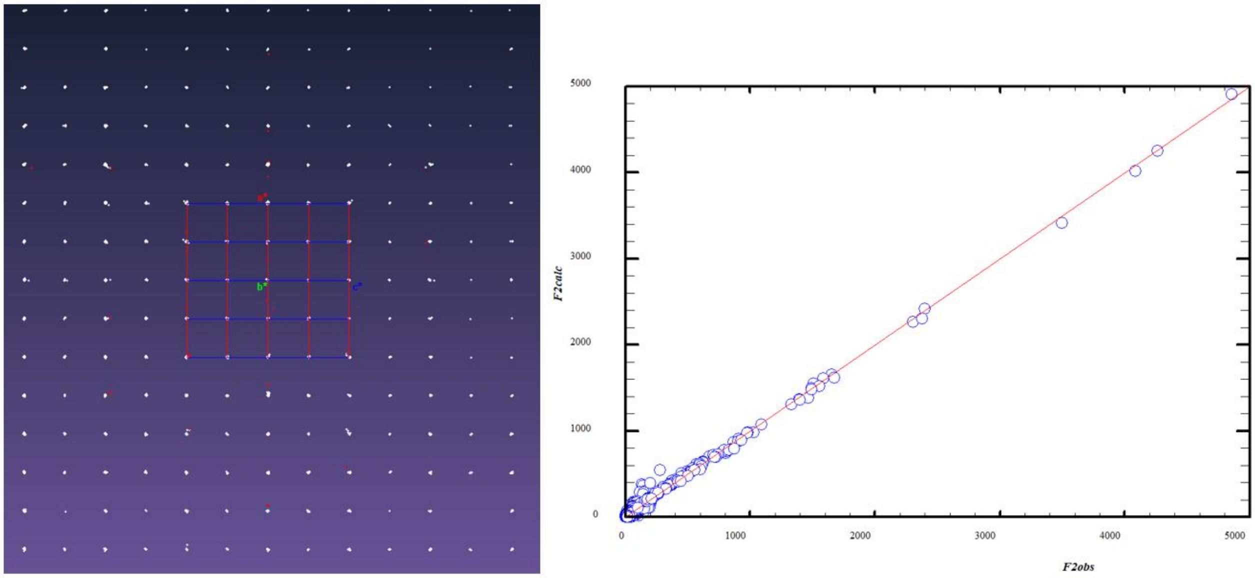

Figure S2: (Left) Single crystal x-ray diffraction pattern at 95 K showing the reflections in the a*c* plane. Red points denote a few unindexed peaks. (Right) Observed and calculated intensity of the single crystal x-ray diffraction at 95 K.

To characterize the crystal, we have collected single crystal x-ray diffraction data at 95 K under N_2 gas flow. With our data collection strategy, we were able to index 99.09% of total 3734 sharp peaks (see figure S2). The as-grown crystal has the $P6_3$ space group which is in agreement with the powder neutron results and the reported structure [1]. All refined structural parameters are tabulated in Table S2 and the refinement of the structure is shown in Figure S2.

Table S1: The structure parameters of $BaCoSiO_4$ measured at 10 K by powder neutron diffraction. The space group is $P6_3$, $a = 9.1124(1)$ Å, $b = 9.1124(1)$ Å, $c = 8.6447(2)$ Å, $\alpha = 90°$, $\beta = 90°$, $\gamma = 120°$. $R_\mathrm{p} = 4.62\%$. $R_\mathrm{Bragg} = 10.5\%$. The atomic displacement parameter B_{iso} is in 1/(8π^2) Å^2.

atom	type	x	y	z	B_{iso}
Ba1	Ba	0	0	0.250	0.22(4)
Ba2	Ba	1/3	2/3	0.2211(9)	0.07(7)
Ba3	Ba	2/3	1/3	0.221(1)	0.25(8)
Co1	Co	0.681(1)	0.676(1)	0.530(1)	0.8(1)
Si1	Si	0.660(8)	-0.0125(7)	0.431(1)	0.35(6)
O1	O	0.7658(6)	0.9118(6)	0.5227(9)	0.48(6)
O2	O	0.4647(5)	0.9029(6)	0.4904(8)	0.28(4)
O3	O	0.7611(7)	0.1927(7)	0.447(1)	0.97(8)
O4	O	0.7245(5)	0.6545(6)	0.7513(9)	1.37(6)

Neutron powder diffraction data collected at 1.8 K using the central wavelength of 2.665 Å were used to determine the magnetic structure without the magnetic field. The magnetic symmetry analysis was performed using the MAXMAGN tool at the Bilbao Crystallographic Server. For a given propagation vector $\mathbf{k} = (1/3, 1/3, 0)$ and the parent grey group $P6_31'$, there are only three k-maximal magnetic subgroups ($P6_3'$, $P6_3$, $P3$) which were tested by comparing the neutron data at 1.8 K. We found the magnetic space group $P6_3$ (No.173.129) that is compatible with a $\sqrt{3}\times\sqrt{3}$ supercell provides a satisfactory result with the agreement factors $R_\mathrm{p} = 5.66\%$ and $R_\mathrm{Bragg} = 18.1\%$. The magnetic model as well as the nuclear phase were refined using the FULLPROF SUITE program. The best refinement and the corresponding magnetic configuration are shown in Fig. S1c and Fig. 2d, respectively. The power-law fit of the intensity of magnetic reflection (2/3 2/3 0) (see main text) in the measured temperature range gives a way to extrapolate the intensity at zero temperature.

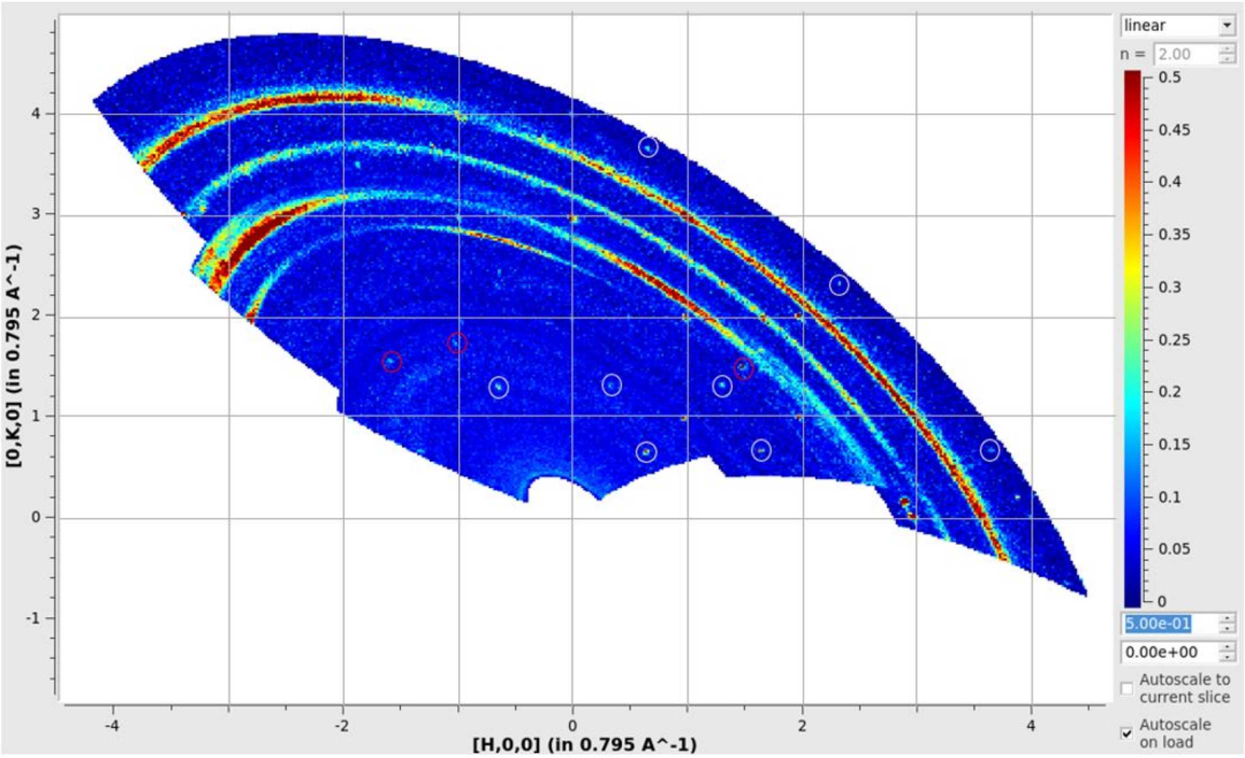

Figure S3: Indexed peaks in the HKL space based on the zero-field single crystal neutron diffraction experiment at 1.5 K in the MAG-B setup. White circles mark the magnetic reflections characterized by the propagation vector **k** = (1/3, 1/3, 0) while red circles denote three spurious reflections which may come from the half-lambda contamination.

Using the refined magnetic moment at 1.8 K, we can calculate the magnetic moment down to zero temperature. The ordered magnetic moment as a function of temperature is shown in Fig. S4a.

The zero-field single crystal neutron diffraction data were collected in a cryomagnet which provides the vertical magnetic field and temperature down to 1.5 K. The purpose of using this setup is to measure the field-dependent neutron diffraction data with a magnetic field along the c axis. Due to the considerable background signals scattered from the facility, the zero-field single crystal neutron diffraction data bear strong rings, as demonstrated in Figure S3. As shown in the figure, we can clearly see the set of magnetic reflections described by the propagation vector **k** = (1/3, 1/3, 0), consistent with the powder neutron diffraction results. Because of this complex sample environment, a decent data reduction for the zero-field single crystal neutron data cannot be obtained precisely. We note that the single crystal neutron diffraction data at 2 T can be reduced and corrected because the propagation vector of the field-induced phase is **k** = (0, 0, 0). The idea is to scale the single crystal neutron data collected at zero field in the cryomagnet with respect to the corresponding reflections from the powder neutron diffraction data. The zero-field single crystal neutron diffraction data are measured with the same sample environment as those at 2 T. Thus, we could do the data reduction and correction of the neutron data at 2T.

The determination of the magnetic structure under 2 T was done based on the single crystal neutron diffraction data at 1.5 K with the field along the c axis using a similar procedure. Given the propagation vector **k = 0** and the parent grey space group, we found two k-maximal magnetic subgroups: $P6_3'$ and $P6_3$. We performed refinement of the magnetic model from the magnetic space group $P6_3$ which is compatible with the neutron diffraction data. Simply consider the symmetry restriction, the former does not support a ferromagnetic component along the c axis, and can also be excluded. Fig. S4b shows the comparison between the observed and calculated neutron diffraction reflections. The best refinement gives the agreement factors $\chi^2 = 16.4$ and $R_\mathrm{F} = 10.5\%$ The corresponding schematic drawing of the magnetic structure is shown in Fig. 2e.

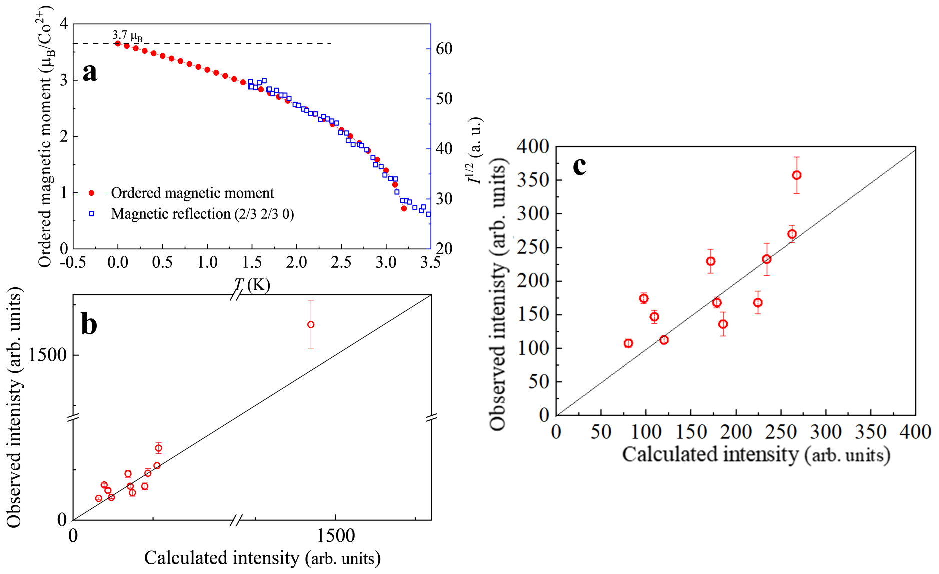

Figure S4: **a**, The square root of the intensity of magnetic reflection $(2/3\ 2/3\ 0)$ and the calculated ordered magnetic moment as a function of temperature. **b**, Observed and calculated intensity of the single crystal neutron diffraction at 1.5 K under 2 T. **c**, Enlarged figure b to show the low intensity neutron data. The error bars are used to show the standard deviation given by the square-root of the number of neutron counts.

Table S2: The structure parameters of $BaCoSiO_4$ measured at 95 K by single crystal x-ray diffraction. The space group is $P6_3$, $a = 9.1225(8)$ Å, $b = 9.1225(8)$ Å, $c = 8.6411(5)$ Å, $\alpha = 90°$, $\beta = 90°$, $\gamma = 120°$. $R_\mathrm{p} = 4.62\%$. The atomic displacement parameter B_{iso} is in $1/(8\pi^2)$ Å^2. Peak hunting yields 3734 peaks in which 99.09% of them were successfully indexed with $R_{int} = 0.03$, $\chi^2 = 4.43$.

atom	x	y	z	B_{iso}
Ba1	0	0	0.25	0.2(1)
Ba2	1/3	2/3	0.221(2)	0.7(2)
Ba3	2/3	1/3	0.223(3)	0.6(2)
Si1	0.674(2)	0.009(3)	0.433(2)	0.3(1)
Co1	0.671(2)	0.683(1)	0.533(1)	0.7(2)
O1	0.768(5)	0.862(5)	0.516(6)	0.4(5)
O2	0.444(5)	0.908(5)	0.490(5)	1.4(7)
O3	0.815(5)	0.246(4)	0.439(4)	0.6(4)
O4	0.746(3)	0.683(4)	0.732(8)	2.8(5)

Table S3: Observed and calculated intensities of the neutron reflections

HKL	F^2_{obs}	F^2_{cal}
0 1 0	224.1196	167.9725
0 2 0	185.4904	135.9625
1 1 0	109.1080	140.5491
-1 1 0	178.5917	167.9725
-1 1 -1	80.0961	107.6398
0 2 -1	119.7928	112.6918
2 1 0	267.3624	357.4929
2 2 0	262.2000	266.8279
2 3 -1	171.8046	229.4886
4 1 0	1423.1833	1649.9768
1 2 0	97.2676	174.3301
0 5 0	233.9305	232.6781

S.2 Magnetization characterization

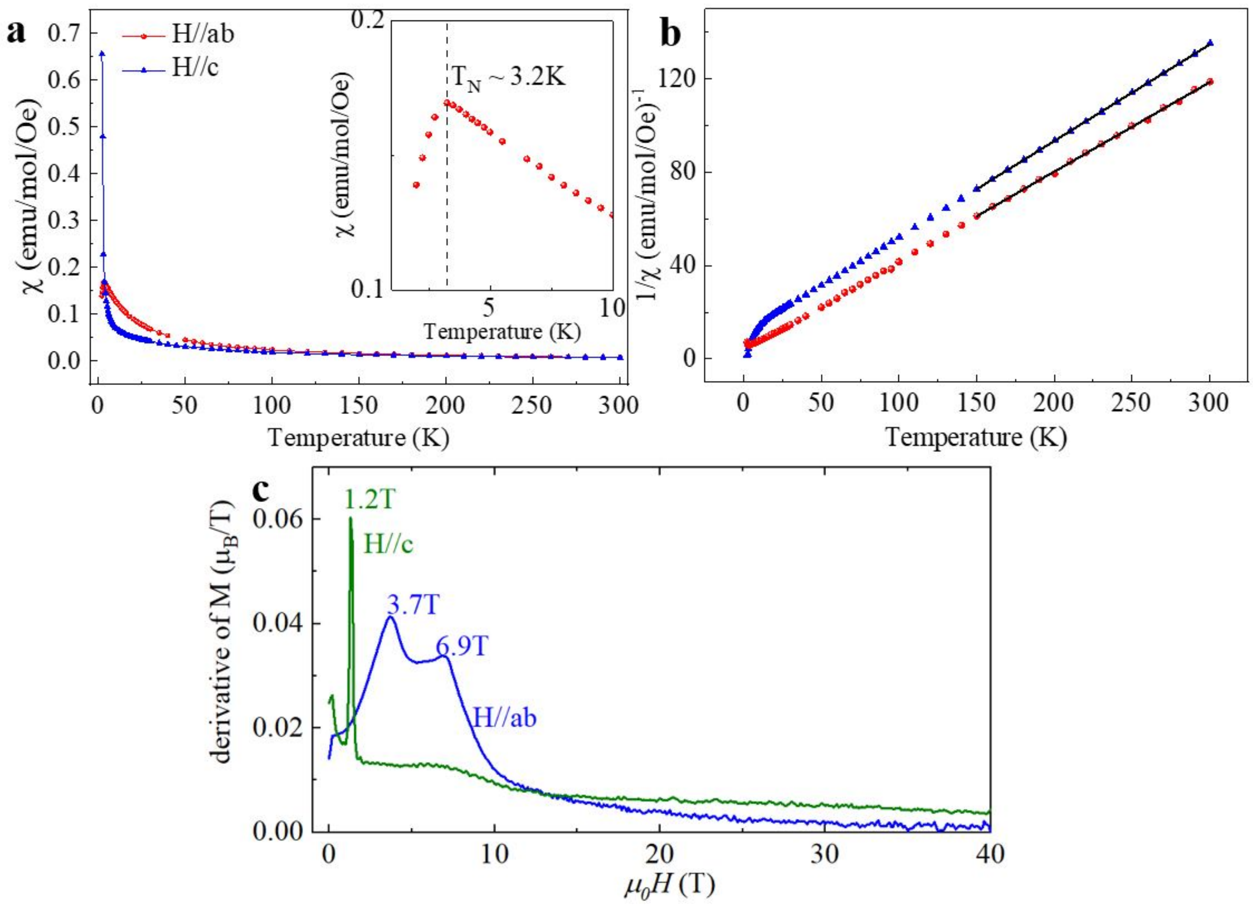

Figure S5: **a**, Temperature dependence of the magnetic susceptibility of $BaCoSiO_4$ with the magnetic field ($\mu_0 H = 0.1$ T) along the c axis and in the ab plane. Inset shows the magnetic ordering temperature. **b**, Inverse magnetic susceptibility curves and the corresponding fits using the Curie-Weiss law. **c**, The derivative of isothermal magnetization curves under magnetic field up to 60 T at 1.5 K.

S.3 Density functional theory calculations

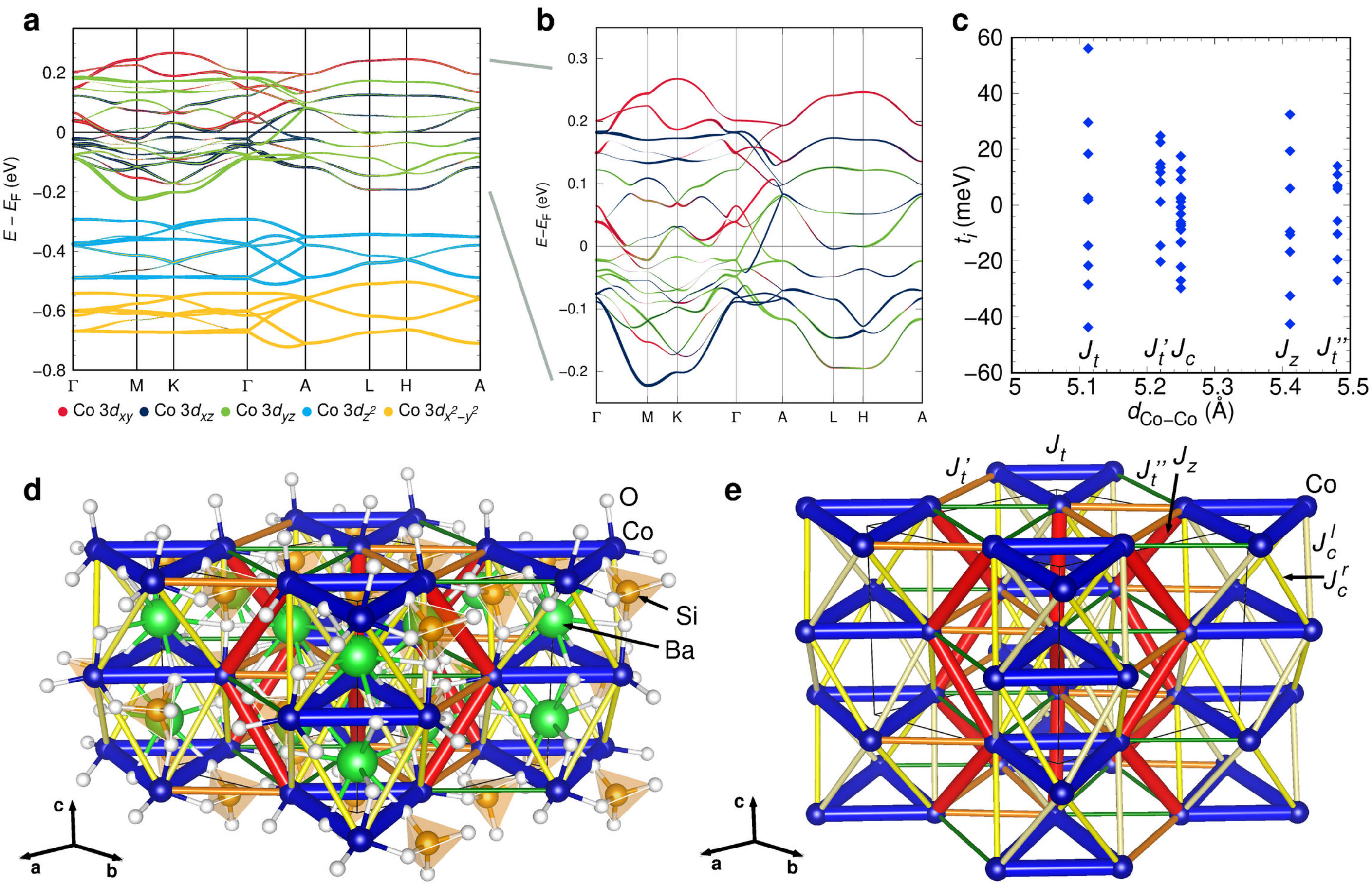

Figure S6: **a** Band structure of $BaCoSiO_4$ with $3d$ orbital character of one Co highlighted, calculated with FPLO basis and GGA exchange correlation functional. **b** Tight binding fit of the 18 t_{2g} bands in **a**. Bands and orbital character fits the DFT result perfectly. **c** Tight binding parameters of the five nearest Co-Co distances. Labels indicate to which exchange path the t_i contribute. **d** Structure of $BaCoSiO_4$ with the first five Co-Co exchange paths drawn as bonds. Bond cross section is proportional to exchange coupling strength. **e** Co network with exchange paths as in **d**.

Electronic structure.- We calculated the electronic structure of $BaCoSiO_4$ using the full potential local orbital (fplo) basis [2] and generalized gradient approximation exchange and correlation functional [3]. Fig. S6a shows the 30 $3d$ bands arising from the six Co^{2+} ions in the unit cell, with $3d$ character of one of the ions highlighted. The local coordinate system is chosen so that the Co-O bonds point into the corners of the cube spanned by the unit vectors. The e_g bands are below the t_{2g} bands and separated from them by a small gap; the t_{2g} bands are half-filled.

Tight binding model.- In order to get an impression which exchange paths might be important, we use projective Wannier functions [4] to obtain a tight binding model for the Co t_{2g} electrons. Bands from this model are shown in Fig. S6b and perfectly match the DFT bands in dispersion and character. Hopping parameters for first five exchange paths are shown in Fig. S6c. These five paths are shown as bonds in Fig. S6d and e. In-plane couplings J_t, J_t' and J_t'' form triangles; inter-layer couplings J_z define zigzag chains along c, and inter-layer couplings J_c are chiral, with J_c^r defining a right screw and J_c^l a left screw; J_c^r and J_c^l belong to the same distance $d_{\mathrm{Co-Co}} = 5.250$ Å but are symmetry inequivalent. The TB parameters do not rapidly decrease with increasing distance, and the inter-layer hoppings ($d_{\mathrm{Co-Co}} = 5.250$ Å and 5.411 Å labeled J_c and J_z) are not substantially smaller than the three in-plane hoppings. On the contrary, there is a suggestion in these parameters that one in-plane coupling (J_t, $d_{\mathrm{Co-Co}} = 5.113$ Å) and one inter-layer coupling (J_z, $d_{\mathrm{Co-Co}} = 5.411$ Å) may play a more prominent role than the other exchange couplings. The conclusion from the tight binding parameters is that it is crucial to calculate all five exchange couplings by energy mapping.

Table S4: Exchange couplings of $BaCoSiO_4$, calculated for a $\sqrt{2} \times \sqrt{2} \times 1$ unit cell within GGA+U at $J_\mathrm{H} = 0.84$ eV and $4 \times 4 \times 4$ k points. The last row contains the Co-Co distances which identify the exchange paths. The errors shown are only the statistical error arising from the energy mapping. The interpolated $U = 4.41$ eV set of couplings (in bold face) reproduces the (average) experimental Curie-Weiss temperature.

U (eV)	J_t (K)	J_t' (K)	J_c (K)	J_z (K)	J_t'' (K)	θ_CW (K)
3	3.48(1)	0.74(1)	0.50(1)	3.03(1)	0.27(1)	-21.8
3.5	3.11(1)	0.64(1)	0.44(1)	2.64(1)	0.22(1)	-19.2
4	2.80(1)	0.54(1)	0.38(1)	2.32(1)	0.19(1)	-17.0
**4.41**	**2.56(1)**	**0.49(1)**	**0.35(1)**	**2.09(1)**	**0.16(1)**	**-15.4**
4.5	2.51(1)	0.48(1)	0.35(1)	2.04(1)	0.15(1)	-15.1
d (Å)	5.113	5.220	5.250	5.411	5.481	

Table S5: Exchange couplings of $BaCoSiO_4$, calculated for a $\sqrt{2} \times 1 \times \sqrt{2}$ unit cell within GGA+U at $J_\mathrm{H} = 0.84$ eV and $4 \times 4 \times 4$ k points for the room temperature structure. The last row contains the Co-Co distances which identify the exchange paths. The errors shown are only the statistical error arising from the energy mapping. The interpolated $U = 4.37$ eV set of couplings (in bold face) reproduces the (average) experimental Curie-Weiss temperature.

U (eV)	J_t (K)	J_t' (K)	J_c^r (K)	J_c^l (K)	J_z (K)	J_t'' (K)	θ_CW (K)
3	3.45(2)	0.73(3)	0.46(3)	0.62(3)	3.03(2)	0.28(3)	-21.6
3.5	3.09(2)	0.62(3)	0.40(3)	0.55(3)	2.65(2)	0.24(3)	-19.0
4	2.77(2)	0.54(2)	0.35(2)	0.50(2)	2.32(2)	0.20(2)	-16.8
**4.37**	**2.57(2)**	**0.49(2)**	**0.32(2)**	**0.46(2)**	**2.11(2)**	**0.17(2)**	**-15.4**
4.5	2.50(2)	0.47(2)	0.31(2)	0.45(2)	2.04(2)	0.16(2)	-14.9
d (Å)	5.113	5.220	5.250	5.250	5.411	5.481	

Energy mapping.- We now extract the Heisenberg Hamiltonian parameters of $BaCoSiO_4$ using the energy mapping technique [5]. We use two different supercells: With a $\sqrt{2} \times \sqrt{2} \times 1$ cell for which the results are summarized in Table S4, we can resolve exchange couplings up to $d_\mathrm{Co-Co} = 9.270$ Å and convince ourselves that only the first five paths shown in Fig. S6e are relevant. Using a $\sqrt{2} \times 1 \times \sqrt{2}$ supercell, with results reported in Table S5, we are able to separate the left-winding and right-winding chiral couplings J_c^r and J_c^l. For the first supercell, the 12 independent Co^{2+} moments in $P\,1$ symmetry allow for 460 unique energies of different configurations; we randomly choose 39 of these spin configurations and obtain an excellent fit to the Heisenberg Hamiltonian in the form

$$H = \sum_{i<j} J_{ij} \mathbf{S}_i \cdot \mathbf{S}_j \tag{S1}$$

Total moments are exact multiples of 3 μ_B as all Co^{2+} moments are $S = 3/2$. In Table S4, the values of the J_i are given with respect to spin operators of length $S = 3/2$. Please note that if the Hamiltonian is written as $\sum_{ij}$ counting every bond twice, the J_i need to be divided by two. Besides the five couplings shown in Table S4, we find negligibly small longer range couplings $J_6 = 0.01(1)$ K ($d_\mathrm{Co-Co} = 7.328$ Å), $J_7 = 0.05(1)$ K ($d_\mathrm{Co-Co} = 7.549$ Å), $J_9 = 0.00(1)$ K ($d_\mathrm{Co-Co} = 8.963$ Å), $J_{10} = 0.00(1)$ K ($d_\mathrm{Co-Co} = 9.084$ Å), $J_{11} = 0.03(1)$ K ($d_\mathrm{Co-Co} = 9.126$ Å), $J_{12} = 0.00(1)$ K ($d_\mathrm{Co-Co} = 9.237$ Å), $J_{13} = -0.01(1)$ K ($d_\mathrm{Co-Co} = 9.270$ Å). The Curie-Weiss temperature estimates are obtained from

$$\theta_\mathrm{CW} = -\frac{2}{3} S(S+1)\Big(J_1 + J_2 + 2J_3 + J_4 + J_5 + J_6 + 2J_7 + J_9 + 2J_{10} + 3J_{11} + 2J_{12} + J_{13}\Big) \tag{S2}$$

where $S = 3/2$. The U value is determined by demanding that the couplings reproduce the experimental Curie-Weiss temperature. From fits to the inverse susceptibility, we have $\theta_\mathrm{CW} = -10(2)$ K for $H \parallel ab$ and $\theta_\mathrm{CW} = -26.2(4)$ K for

$H \parallel c$. As approximate energy scale, we use a weighted average of these two values, $\theta_{\rm CW} = -15.4\,\rm K$. The corresponding interpolated set of exchange couplings is given in Table S4 in bold face.

For the second supercell ($\sqrt{2} \times 1 \times \sqrt{2}$), the 12 independent Co^{2+} moments in $P\,1$ symmetry lead to 195 spin configurations with distinct energies out of which we use 38 for the energy mapping. Besides the six couplings given in Table S5, we obtained negligibly small $J_8 = 0.05(1)\,\rm K$ ($d_{\rm Co-Co} = 8.683\,\rm Å$) and $J_{14} = 0.01(1)\,\rm K$ ($d_{\rm Co-Co} = 10.076\,\rm Å$). The two chiral couplings J_c^r and J_c^l turn out to be substantially different, with J_c^r 50% larger than J_c^l.

Fully relativistic calculations.- We use collinear relativistic DFT calculations to estimate the single ion anisotropy $E_{\rm SIA} = AS_z^2$. In order to separate $E_{\rm SIA}$ from anisotropic exchange, we calculated total energies for three different spin configurations for the six Co ions in the unit cell: a) FM (ferromagnetic). b) TAFM (one J_t triangle up, one J_t triangle down). c) STRIPY (up-up-down in one J_t triangle, up-down-down in the other). We calculated energies for moments $\mathbf{m} \parallel \mathbf{x}$, $\mathbf{m} \parallel \mathbf{y}$, and $\mathbf{m} \parallel \mathbf{z}$; the first two energies are the same, the third is higher.We use both plain GGA+SO calculations and GGA+SO+U with $U = 4\,\rm eV$ (the value we know to describe the material correctly). The single ion anisotropy energy estimates are listed in Table S6. The fact that the dependence on spin configuration is small (only about 10% variation) indicates that anisotropic exchange is not strong.

Table S6: Estimates of single ion anisotropy energies A for three spin configurations.

	$A_{\rm FM}$ (K)	$A_{\rm TAFM}$ (K)	$A_{\rm STRIPY}$ (K)
GGA+SO	1.85	1.85	1.82
GGA+SO+U ($U = 4\,\rm eV$)	2.38	2.14	

S.4 Theoretical modeling

Consider one triangle with three spins coupled by the DM interactions that respect the 3-fold rotation symmetry. The total energy is given by $E^{\text{tot}}_{\text{DMI}} = (\mathbf{S}_1 \times \mathbf{S}_2) \cdot \mathbf{D}_3 + (\mathbf{S}_2 \times \mathbf{S}_3) \cdot \mathbf{D}_1 + (\mathbf{S}_3 \times \mathbf{S}_1) \cdot \mathbf{D}_2$, where spins $\{\mathbf{S}_i,\ i = 1, 2, 3\}$ are numbered counterclockwise on the triangle, and $\mathbf{D}_i$ is the DM vector on the opposite edge of $\mathbf{S}_i$. The energy associated with the out-of-plane component of the DM vectors is $E^{\text{z}}_{\text{DMI}} = D_z \hat{z} \cdot (\mathbf{S}_1 \times \mathbf{S}_2 + \mathbf{S}_2 \times \mathbf{S}_3 + \mathbf{S}_3 \times \mathbf{S}_1) \equiv D_z \hat{\mathbf{z}} \cdot \boldsymbol{\epsilon}$, where $\boldsymbol{\epsilon}$ is the vector spin chirality. This term always favors coplanar spin configurations, for which $|\hat{\mathbf{z}} \cdot \boldsymbol{\epsilon}|$ is maximized (Fig. S7).

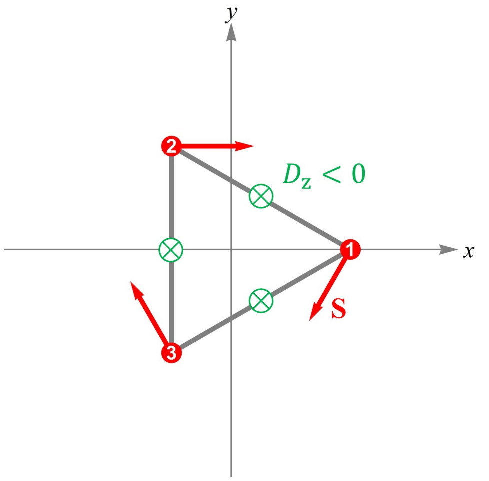

Figure S7: Minimal energy configuration for $D_z < 0$ (Left) and $D_z > 0$ (Right). A uniform rotation applied to all spins does not change energy.

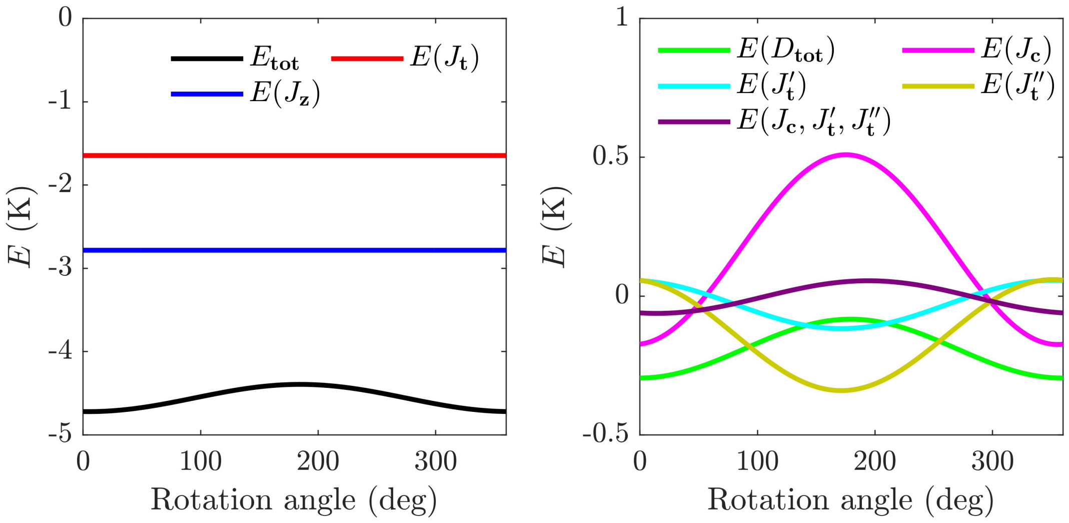

Figure S8: Energy per spin for each of the terms in the Hamiltonian as a function of rotation angle.

Focusing on spin configurations with the 3-fold rotation symmetry, we derive the total DMI energy with a spin and the DM vector on its opposite edge written in the same local frame, $\mathbf{V}_i = V_z \hat{\mathbf{z}} + V_\perp(-\hat{\mathbf{r}}_i) + V_\parallel \hat{\mathbf{z}} \times (-\hat{\mathbf{r}}_i)$ where $V \equiv S$ or D [Fig. 3a], and $\hat{\mathbf{r}}_i$ is the unit vector from the center of the triangle pointing to site i. The result is

$$E^{\text{tot}}_{\text{DMI}} = \frac{3}{2}\sqrt{3} D_z \left(S_\parallel^2 + S_\perp^2\right) + 3\sqrt{3} S_z (D_\parallel S_\parallel + D_\perp S_\perp) \equiv \frac{3}{2}\sqrt{3} D_z |\mathbf{S}_{xy}|^2 + 3\sqrt{3} S_z \mathbf{S}_{xy} \cdot \mathbf{D}_{xy}\,, \tag{S3}$$

where $\mathbf{S}_{xy} = (S_\perp, S_\parallel)$ with the $\mathbf{S}_i$ component parallel to radius-vector $\mathbf{r}_i$ as $S_\parallel$ and that perpendicular to it $S_\perp$, and $\mathbf{D}_{xy} = (D_\perp, D_\parallel)$. Other relevant physical quantities can be calculated: toroidal moment $\mathbf{t} = \sum_i \mathbf{r}_i \times \mathbf{S}_i = 3S_\perp \hat{\mathbf{z}}$; vector spin chirality $\boldsymbol{\epsilon} = \mathbf{S}_1 \times \mathbf{S}_2 + \mathbf{S}_2 \times \mathbf{S}_3 + \mathbf{S}_3 \times \mathbf{S}_1 = \frac{3\sqrt{3}}{2} S_{xy}^2 \hat{\mathbf{z}}$; scalar spin chirality $\kappa = (\mathbf{S}_1 \times \mathbf{S}_2) \cdot \mathbf{S}_3 = |\boldsymbol{\epsilon}| S_z$.

To understand the role played by the subleading interactions, we start with the energy-minimized ferritoroidal structure in zero field, continuously rotate along the c-axis all spins on the sublattice with toroidal moment opposite to the net moment and plot the energy of each term in the Hamiltonian as a function of rotation angle in Fig. S8. The left panel shows the total energy (black), the energy of J_t bonds (red) and that of J_z (blue). Since the global rotation occurs within one of J_t-J_z sublattices, the energy of these two bonds stays the same. The right panel of Fig. S8 shows the energy of all subleading interactions. It is evident that the J_c term favors the ferritoroidal state, while J_t' and J_t'' favors the ferrotoroidal one.

References

[1] Liu, B. & Barbier, J. Structure of the stuffed tridymite derivatives, Ba*M*SiO_4 (*M* = Co, Zn, Mg). *J. Solid State Chem.* **102**, 115–125 (1993).

[2] Koepernik, K. & Eschrig, H. Full-potential nonorthogonal local-orbital minimum-basis band-structure scheme. *Phys. Rev. B* **59**, 1743–1757 (1999).

[3] Perdew, J. P., Burke, K. & Ernzerhof, M. Generalized gradient approximation made simple. *Phys. Rev. Lett.* **77**, 3865–3868 (1996).

[4] Eschrig, H. & Koepernik, K. Tight-binding models for the iron-based superconductors. *Phys. Rev. B* **80**, 104503 (2009).

[5] Iqbal, Y. *et al.* Signatures of a gearwheel quantum spin liquid in a spin-1/2 pyrochlore molybdate Heisenberg antiferromagnet. *Phys. Rev. Mater.* **1**, 071201(R) (2017).
